# Hip Dislocations

**Published:** 1870-01

**Authors:** Henry J. Bigelow

**Affiliations:** Boston


### HIP DISLOCATIONS.—REPLY OF PROFESSOR BIGELOW TO PROFESSOR GUNN.

Mr. Editor:—In a pamphlet* which Professor Gunn has somewhat widely distributed in this vicinity, and which has been brought to my notice since I recently returned from abroad, he makes the following remarks:—

"Professor Bigelow does me great injustice in the manner in which he alludes to my writings; on this subject, he says:—

"'Professor Gunn maintains, in a paper upon this subject, that any untorn or undissected portion of the capsular ligament is capable of producing the signs of hip and shoulder luxations.'"

This is quoted from a part of my paper, intended to do justice to the theories of different writers, upon the questions of muscular ard capsular resistance, in hip dislocation.

I trust that Professor Gunn, for whom I entertain great respect, will acquit me of any intentional act of injustice. The above statement was made upon a careful study of his pamphlet, which I first met with several years after my own theory was made public. In now reëxamining Professor Gunn's pamphlet, I see no reason for modifying the statement to which he objects; indeed, his views differ so widely from mine, in respect to the varieties, classification, pathology, and treatment of hip dislocation, and, in fact, as to everything, except the general region of the capsule, concerned in two of seven regular dislocations, that his pamphlet does not strictly require a reply.

Professor Gunn confines himself chiefly to the question of resistance to reduction, in two dislocations, namely, the dorsal and so-called "ischiatic." It may be observed, however, that

* Luxations of the Hip and Shoulder Joints, and the Agents which Oppose their Reduction. By Moses Gunn, A.M., M.D., Professor of the Principles and Practice of Surgery and Clinical Surgery, in Rush Medical College; formerly Professor of Civil and Military Surgery, in the University of Michigan. Second Edition. Chicago, 1869.

of the two dislocations alluded to, he fails to appreciate the character of what he, in common with previous writers, calls the "ischiatic" luxation, which, in the only case he cites (p. 13), was, as he says, primarily dorsal. If it was primarily dorsal, as alleged, Professor Gunn's remarks really refer, not to two luxations, but only to one, namely, the dorsal; because, as the tendon of the obturator internus must have been ruptured, to allow a change of place from the dorsum to the ischiatic notch, the luxation he calls "ischiatic" had no proper claim to be so designated, but was simply dorsal. But the present communication is addressed only to the question of "injustice" to Professor Gunn.

My paper † embraces a general treatise upon hip luxations, of which seven regular varieties are enumerated, four, at least, being new, and all owing their phenomena, as I believe, chiefly to a single portion of the capsule of the hip, namely, to the ilio-femoral or Y ligament (so called for brevity), in one or both of its branches.

Prof. Gunn, on the other hand, recognizing only the four luxations of previous writers, urges that, in a paper published in 1853, and subsequently incorporated with other papers in a pamphlet which he now republishes, he had already identified the *anterior and inferior portion* of the capsular ligament as a cause of resistance to reduction in the *dorsal* luxation, and in the (erroneously so called) *ischiatic* luxation. But he fails to add, that he similarily identifies, in the same pamphlet, *another and different part* of the capsule as the cause of the resistance, in *other* luxations.

Again, Professor Gunn states, that in the "dorsal" and so-called "ischiatic" luxations (after the posterior and upper half of the capsule is cut away), the *anterior and inferior half* of the capsule binds down the head of the bone, and opposes its reduction (p. 5).

† The Mechanism of Dislocation and Fracture of the Hip. With the Reduction of the Dislocations by the Flexion Method. By Henry J. Bigelow, M.D., Professor of Surgery and Clinical Surgery in the Medical School of Harvard University; Surgeon of the Mass. Gen. Hospital, etc. Philadelphia, 1869.

Professor Gunn here speaks only of two similar and allied luxations—probably only of one—and shows no knowledge of the existance of an ilio-femoral ligament. He also points out, as essential, the resistance of the inferior portion of the capsule, adjoining the obturator foramen, where it is actually thinest and least capable of resistance to reduction. Indeed, he defines very imperfectly even the "half" of the capsule to which he calls attention. In an account of an experiment in the winter of 1858–59, he says (p. 11), that "the upper and *posterior* half of the capsule was then cut away;" while in reference to other experiments, in 1868–9, he says (p. 18), that "the upper and *outer* portion of the capsular ligament" was removed, at the same time identifying this as "the dissection used in former experiments."

But I am nevertheless ready to concede that, within these narrow limits, namely, in designating loosely *an anterior capsular resistance in dorsal dislocation*, Professor Gunn is, in the main, correct and deserving of the credit it was my object to accord to him. He goes further, and, as I think, astray.

He directs that, "*in the forward dislocation upon the pubes*, while extension and counter-extension is being made in the usual manner" (*i.e.*, by the old method of longitudinal extension), "the limb should be rotated externally; this," he says, "relaxes *the posterior and untorn portion* of the ligament" (p. 20).

I think that Professor Gunn will not contend that this *posterior* portion of the capsular ligament, "untorn," as he says, in the pubic luxation, and, therefore, to be relaxed by a special position of the limb, is identical with the *anterior* portion, which he has repeatedly before pointed out as "untorn," in the dorsal luxation, and which is to be relaxed, as he states, by a wholly different position of the limb.

It is plain that *two different parts* of the capsule are here referred to by Professor Gunn; and as these two parts manifestly comprehend the whole, these passages alone are sufficient to show that there is no injustice in attributing to him, in the sentence he has quoted, the theory that "*any* untorn or undissected portion of the capsular ligament is capable of producing the signs of hip and shoulder luxations."

But in thus refuting the specific charge of Professor Gunn, I have not spoken of the general drift of his whole pamphlet, the conclusions of which are summed up on its last page, and which still further substantiate my expressed conviction. His theory for both hip and shoulder is, that, when the bone passes to one side of the socket, it tears that side of the capsule, and leaves the rest sound, and that the essential resistance to reduction is due to this remaining sound side. This theory is, indeed, the key-note of Professor Gunn's paper, and upon it he basis his system of reduction. In the dorsal dislocation, for example, of which it almost exclusively treats, he considers that because the head of the bone is found upon the dorsum, it should be reduced through the dorsal laceration, and reiterates (pp. 13, 14, 16, 18, 20), even in italics, the necessity of *adducting* the limb across the other for this purpose. In his only reported case, he accomplishes this (in his own words, "*arte non vi*"), by a Jarvis' adjuster (p. 13). He says (p. 20), "We further lay down the following special rules:—In the luxation upon the dorsum ilii, the patient lying on his back, carry the limb across its fellow, at a point corresponding with the union of the middle with the upper third, rotate inwards, and the pelvis being fixed by an assistant, the head may now be readily drawn into its place;" that is, through the dorsal laceration of the capsule, no matter how inconsiderable this may be. According to my theory, dorsal dislocations are best reduced from below the socket, after the capsular orifice is enlarged, if it is constricted, with the thigh flexed at right angles, to relax the Y ligament, and, sometimes, even finally *abducted*.

"In all dislocations," says Professor Gunn, finally, "place the limb in just the position which characterized it at the moment of escape, and the reduction will then be easily effected" (p. 20); a direction which would prove, I think, sometimes impracticable and sometimes erroneous. Different positions for the different luxations, both of the hip and shoulder, are enumerated in "special" and "general" rules (pp. 19–20); clearly indicating a belief, that in different luxations, *different parts of the capsule are torn*, and, as a consequence, that *different parts of the capsule remain*, at once essential to the phenomena, and requiring to be relaxed by wholly different positions, in order to reduce the limb.

My meaning would, perhaps, have been more exactly rendered by saying, "Professor Gunn maintains, in a paper upon this subject, that *different* (rather than *any*) untorn or undissected portions of the capsular ligament are the essential agents of resistance in the reduction of hip and shoulder luxations."

*This doctrine is wholly opposed to my own*, which recognizes the Y *ligament* as the chief obstacle to reduction, in seven regular dislocations, and directs flexion of the thigh to overcome its resistance.

But upon this point, Professor Gunn, after speaking of the "anterior and inferior portion," or, as he also characterizes it, "half," of the capsular ligament, and its relation to dorsal dislocation, says: "Surely this language includes the so-called Y ligament, unless its author finds also a new location for that structure;" thus claiming, by implication, a knowledge of the pathological functions of the Y ligament. Professor Gunn should remember, that although the greater may, in one sense, include the less, as a block of marble does a statute, yet that there is no sentence or word in his whole pamphlet which alludes to the existence of an ilio-femoral ligament in the capsule. Inasmuch as this ligament is one of the strongest in the body, "thicker than the ligament of the patella or of the tendo-Achillis," § and is only a part of the literally "undissected" (p. 19) half designated by Professor Gunn, as producing the phenomena of dorsal dislocation, it is difficult, in view of this silence, to understand how it could have been in his mind, at the time of writing his pamphlet, or why, in common with all other modern surgical writers and authorities, he should have wholly ignored it in this relation, till after my paper was published.

Since the above was written, my attention has been directed

§ Traité d'Ostéologie, etc., S. P. Soemmering and G. & E. Weber. Paris, 1843. Pp. 323–324.

to a notice in the *Chicago Medical Journal*, (October, 1869, p. 881), reiterating, under the term "unfairness," Professor Gunn's charge of "injustice." The writer says also: "So far as we are able to see, this author (Professor Bigelow) has added nothing to Professor Gunn's exposition on the subject, save only this new name of that portion of the capsular ligament—the anterior and inferior—heretofore known as the ilio-femoral, but which he christens as the Y ligament."

This zealous defender of Professor Gunn obviously possesses one alleged qualification of an impartial critic: he has not read the paper he criticizes. His remarks require notice only so far as they relate to the charge of "unfairness," which, I think, needs no further comment.

HENRY J. BIGELOW.

BOSTON, *Nov. 24, 1869.*

