# Erratum

**Published:** 1870-03

**Authors:** 


PROFESSOR BIGELOW'S LETTER IN OUR LAST NUMBER.—The following is from the *Boston Medical and Surgical Journal*, Jan. 27.

ERRATUM.—The word "published"* was here accidentally substituted, in the printed proof, for *made public.* By seeming to refer to the *printing* of my paper in 1869 instead of its *public reading* in 1861 (see p. 10), it does injustice to certain European observations in 1865–8; and on this account I am unwilling to leave it uncorrected. But this is a separate and here wholly incidental question. As far as the passage relates to Prof. Gunn, I believe it to be strictly correct as it stands. I cannot find that he alludes to the ilio-femoral ligament before Sept. 17th, 1869. H. J. B.

*At the end of the last paragraph but two.

